# Topics of Public Interest

**Published:** 1904-10

**Authors:** 


### TOPICS OF PUBLIC INTEREST.

### Why We Did Not Unite.

(*From New York State Journal of Medicine, September, 1904.*)

The following is a copy of the affidavit submitted by the Onondaga County Association in opposition to the motion to amalgamate the two state organisations.

The part which is of special interest is that referring to the notice of meeting at which the state association instructed the committee to proceed in bringing about the amalgamation and voting amalgamation for the state organisation. It appears that in the state by-laws there is no provision for sending notices of meetings, and there are decisions in our state which provide that in this contingency *personal* notice must be sent to *each member* of a membership organisation like the state association, giving the day, place and hour, and the purposes for which the special meeting is called. This personal notice cannot be given by mail, but must be *served personally upon every individual* person in the association; if one were omitted, that one would have the right to upset the entire plan should he remain away from the meeting.

It appears, therefore, that the action of the state association in March in voting property rights or vested rights of individuals to which the above rules apply, is a nullity, and the result attained of no binding force on the members of the state medical association. This is the situation of affairs at present.

SUPREME COURT.

In the Matter of

The Application of the Medical Society of the State of New York and The New York State Medical Association, for an order consolidating the said corporations, pursuant to the Act, Chapter 1, of the Laws of 1904.

STATE OF NEW YORK,
County of Onondaga, } ss.
City of Syracuse,

GEORGE A. EDWARDS, being duly sworn, deposes and says, in answer to the petition of the petitioners herein, that he is a duly licensed physician and surgeon of the state of New York, and resides at the city of Syracuse, Onondaga county, in said state; that he has practised his profession continuously in said city for more than twenty-seven years; and ever since the organisation of The New York State Medical Association has been and now is a member thereof; that he is also a member of the county association of Onondaga county, and is at present an officer thereof, namely, vice-president; that he is also a member of the Medical Society of the County of Onondaga, but not a member of the Medical Society of the State of New York; that he has read the moving papers herein and knows the contents thereof; that deponent believes it would be unwise to consolidate the New York state medical society and the New York state medical association pursuant to the agreement attached to the moving papers herein, for the reason that the said association has vested property rights aggregating over $30,000 in value in excess of that of said society, which property is the property of the 1,767 physicians composing the said association; that said 1,767 physicians should not suffer a usurpation of their property rights by compelling a division of the assets of the association among the 5,733 composing the state and county medical societies of the state of New York. And, furthermore, the members of the state association are entitled to their personal rights and should have the privilege of maintaining the liberal ethical code under which they have for years practised, which is the code of the American Medical Association. That, by the terms of the proposed agreement said ethical code is entirely surrendered, and the transformed society, until a vote in reference thereto is taken, will be without any code whatever. That said agreement does not contain a complete plan for the adoption of a code, as it makes provision only for the taking of a vote concerning the same. That the code of said association should have been embodied in this agreement, for the reason that it is the code approved by the medical association and must necessarily be maintained if the members of the state society are to become members of the said American Medical Association; but, whether the code of said state association should be adopted, or that of the medical society, there should, at all events, have been in said agreement some ethical code which should have been submitted to all the members of both the state society and the state association and approved by at least a three-fourths vote of all said members at a meeting of said society and association called for that purpose, as provided by section 7 of the membership corporation law of the state of New York. That, to carry out the plan embodied in the agreement will be, in the opinion of deponent, very prejudicial to physicians, and it might result in greater cleavage than has heretofore existed among the members thereof. Deponent believes that before an order is made consolidating the petitioners herein, all the terms should be agreed upon and not left for further consideration by the members of the profession.

And deponent further says, that said agreement is further objectionable since it not only provides that the property of the said state association shall practically be confiscated, but also because it provides that all county associations shall transfer their property and assets to the county society of the same county. That said county associations are independent of the state association, said state association having no control whatever over the assets of any county association, although no physician can become a member of the state association without having been voted in by the county association.

And deponent further says, that, personally, he has never received any notice of a special meeting of the state association called for the purpose of passing on the proposed agreement attached to the moving papers herein. That the by-laws of the state association contain no provision for giving notice of annual or special meetings of the association; and, while deponent has not seen the secretary's minutes, yet he is informed and believes that no notice has been given to the physicians generally who are members of the said association.

And deponent further says, that if the members of the medical profession are desirous of forming a union, in fairness to the members of The New York State Medical Association, the charters of both the New York Medical Society and The New York State Medical Association should be surrendered and their corporate affairs wound up. That, by the present plan, there is a swallowing up, or absorption of The New York state medical association, having assets of over $34,000, with an annual income of $12,000, and a surrendering of its ethical code in addition. That deponent believes the only reason why the state medical society has desired to form a union with The New York state medical association is in order that its members might become affiliated with the American Medical Association; that having such meager assets and a rapidly declining membership, a few of the more potential ones, wishing to escape humiliation and disgrace, devised this plan of gaining membership in the New York state association without resigning from membership in the New York state medical society.

And deponent further says, that he believes the special law passed in 1904 for the purpose of allowing a consolidation of the New York state medical society with that of The New York state medical association is unconstitutional; and that, without the unanimous consent of all the members of said state association, it will be a usurpation of property rights to grant an order consolidating said corporation; that deponent personally objects to such consolidation; and, furthermore, the Onondaga County medical association also objects to such consolidation, and has voted unanimously to oppose this application.

George A. Edwards.

Sworn to before me this 16th day of

July, 1904.

Chas. C. Cook,
Com'r of Deeds, Syracuse, N. Y.

---

### Council Meeting.

*(From New York State Journal of Medicine, September, 1904.)*

A meeting of the council of The New York State Medical Association was held August 29. The following letter was received from the counsel to the association, and ordered printed in the *Journal:*

———, August 23, 1904.

Dr. William Harvey Thornton, President, and Council The New York State Medical Association:

*Gentlemen*—By reason of the physical impossibility of my being present at the meeting of the council to be held on the 29th, of which I have just this moment received notice, and as I desire to apprise The New York state medical association, through its council, of the exact status of its affairs with reference to the proposed amalgamation, I beg leave to submit the following:

During the session of the legislature of this state last winter, an act was passed authorising the New York State Medical Association and the Medical Society of the State of New York, under certain conditions to unite. Pursuant to the permission granted by this act a special meeting of The New York State Medical Association was called for March 21st, notice whereof having been mailed, stating the purpose of the meeting, to the last known address of each and every member of The New York State Medical Association. At this meeting a resolution was offered and adopted indorsing a plan of uniting the two state medical organisations.

Subsequently, armed with the resolution adopted at this special meeting of the New York State Medical Association, and with a similar resolution adopted by the Medical Society of the State of New York, application was made to the Supreme Court of the County of New York for an order joining the two organisations into one body. The application for this order was made returnable in the Borough of Manhattan, before Mr. Justice Fitzgerald, and upon the return day an affidavit was submitted in opposition to the application, sworn to by the vice-president of the Onondaga County Medical Association, which affidavit, among other things, raised the question as to whether or not a sufficient notice of the special meeting of March 21st had been given to each and every member of the state medical association, to make the particular resolution adopted at that special meeting a valid one binding upon each individual member of the state medical association.

Upon examination of the by-laws of the New York State Medical Association it then appeared that there was no by-law providing for the manner of giving notice of meetings of the association.

The various decisions in this state seem to indicate that where the manner of giving notice of meetings of membership corporations is omitted from the by-laws, then the common-law rule applies which requires that if resolutions are to be passed at any meeting which in any manner involves any personal privilege or rights of person or property acquired by membership in such association, that this notice of the meeting must be served upon each individual member personally, not through the mail nor by word of mouth, and such notice must contain the date, place and hour of the proposed meeting and the purpose for which it is called. Ordinary business meetings, where no such privileges or rights are involved, I believe would not require such formality.

It would seem, therefore, that the resolution in favor of amalgamation adopted at the meeting held on the 21st day of March last, in so far as it binds the members of The New York State Medical Association to a plan of amalgamation with many other organisations, and in so far as it effects the privileges acquired by membership, and the rights of person or property thus acquired, was null and void and had no effect, and there would appear to be left open but two courses; one is to call a special meeting after such personal notice as I have indicated has been served upon each individual member of the association, which seems well-nigh impossible to perform; the other one is to offer at the October meeting a resolution designating the manner of serving notices upon members of the association, which resolution will stand over for one year when action may be taken anew.

Respectfully yours,

JAMES TAYLOR LEWIS.

Counsel, The New York State Medical Association.

Dr. Townsend offered the following resolution, seconded by Dr. Brown and immediately carried:

WHEREAS, It appears that there are inconsistencies in the by-laws of The New York State Medical Association, and

WHEREAS, It appears that one of the causes of the failure of the application for amalgamation of the two state organisations was due to insufficient notice being served upon members of the association, in a measure owing to the fact that provision for the service of notice upon members by mail was omitted from the by-laws of the state medical association.

*Resolved,* That the special committee on revision of the by-laws, with the assistance and coöperation of the counsel of the New York State Medical Association, be requested to submit for action at the annual meeting in October, 1904, by-laws covering the various deficiencies and uncertainties existing in the by-laws of the New York State Medical Association, and that the secretary be instructed to forward a copy of this resolution to the chairman of the special committee appointed upon a revision of the by-laws.

---

### The N. Y. S. M. A.

(*Editorial New State Journal of Medicine, September, 1904.*)

SINCE, practically, the only object in the uniting of this association and the society of this state was the preservation of the name of the state society, which demanded not a little respect on account of its hoary locks, and since all the members of the medical profession in this state wish to see harmony and a united front, and since the New York State Medical Association is the body which is affiliated with and a part of the American Medical Association; and since the constitution which the new society was to have adopted is the same, or nearly the same, as the present one of the association, and much improved over the constitution of the society; and since the medical profession as a whole, through committees and elections and votes, and in every way possible, has expressed a desire to have one society in this state, which should be the representative of the American Medical Association, which should have a constitution and by-laws drawn up according to the form recommended by the American Medical Association, and is already adopted by most of the states. Also to preserve the name of the Medical Society of the State of New York.

Now that this scheme has failed, how can the profession most necessarily and readily fill its wants?

The New York State Medical Association, as it exists today, has all these advantages, and many others, to offer its members, with the exception of the name.

It would be nice to have the age of a century, it certainly lends dignity; but if we can't have it except by joining, why, we'll get it that way.

The plan of the organisation in this state, as drawn up, and followed by the New York State Medical Association, is followed now by the American Medical Association and the various states. The protection in malpractice suits, afforded to the members by our legal department, is highly commented upon in the lay press, and is being copied in other states with equal success.

Our directory is the only one which is exact (as far as human care can make it) in this state, and has been called by extensive advertisers the best compiled medical directory in the country.

Our *Journal* is an assured success, and this method of publishing transactions, instead of in the bulky annual order, has been copied in several states, and is soon to be begun in others. In truth, we see no object in dissolving both medical association and the medical society of this state. By so doing we would not gain the advantage of the age of the state society. Necessarily, it would be lost; and what is there left to gain? Everything else is possessed now by the New York State Medical Association. Those who wish these advantages—and we believe all do—can join.

The right hand of fellowship we hold out to all physicians in good standing.

Let those who are jealous to preserve the more aged society belong to both. Preserve the other if you will, by a meeting now and then—though, for ourselves, we are not antiquarian (some of us).

We want a united front, we of the medical profession of New York State, and rather than go to further expense and trouble, and take a larger time to accomplish what can be accomplished immediately, except for the promulgation of an ancient and honorable name, let those who have been waiting now come forward and join the association. What is the use to any one of further litigation? The law has decided that what we all wanted and wished for is impossible. Let us accept the next best plan, and with love and affection, and faithfulness to the old society, by its members who cherish its memory—let us all, every one, come together in The New York State Medical Association.

